# Supplementary material for: A Small-Angle Neutron Scattering, Calorimetry and Densitometry Study to Detect Phase Boundaries and Nanoscale Domain Structure in a Binary Lipid Mixture
Source: Membranes (Basel). 2023 Mar 10;13(3):323. doi: 10.3390/membranes13030323 (PMC10051979; doi:10.3390/membranes13030323)
Supplement: Supplementary file 1 [file membranes-13-00323-s001.zip › membranes-2252864-supplementary_revised.pdf]

Supplementary Material for:

A small angle neutron scattering, calorimetry and densitometry study to detect phase boundaries and nanoscale domain structure in a binary lipid mixture.

Natalie Krzyzanowski, Lionel Porcar, Ursula Perez-Salas

S1 Vesicle extrusion

The Mini-Extruder (set) with heating block from Avanti Polar Lipids was modified as follows:

S1.1 Metal cylinder and sealing of membrane filters

Avanti's 2-cylinder Teflon construct, which sandwiches the membrane filter and supports, is encased in a hollow metal cylinder with an open screw top which pressures the pieces together. We modified this cylinder by making it longer so that both Teflon cylinders fit in the hollow region of the metal cylinder and thus avoid the Teflon pieces from deforming, particularly for extrusions that required heating. We added a metal disc provides uniform pressure to the encased Teflon. A holder, in the form of a spoke on a base, places the first Teflon piece near the edge of the hollow cylinder (see Figure S1.1) for easy placement of membrane supports and membrane filter and the proper placement and alignment of the second Teflon piece. Once the second Teflon piece is set in place over the first piece of Teflon – o-rings facing each other – the 2-cylinder Teflon construct can slide down the hollow cylinder by simply lifting the metal cylinder out of the spoke. A small gap above the 2-cylinder Teflon construct allows for the secure placement of the metal disc, which is then pressed by the screw cap.

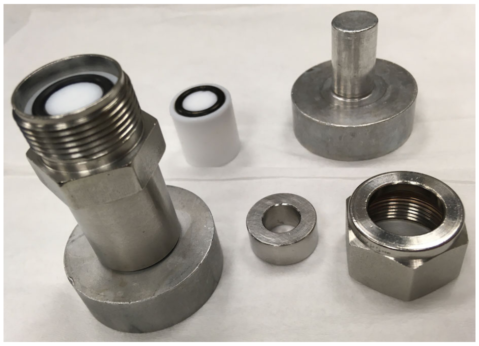

Figure S1.1 Shown metal cylinder over spoke on a base with first Teflon piece inserted into the cavity of the cylinder. The Teflon piece is near the edge of the hollow cylinder which makes the placement of membrane supports and membrane filter and the proper placement of the second Teflon piece over the first easier. Lifting the cylinder from the base lets the two Teflon pieces fully slide into the cavity. Metal ring and screw cap uniformly press the o-rings to seal.

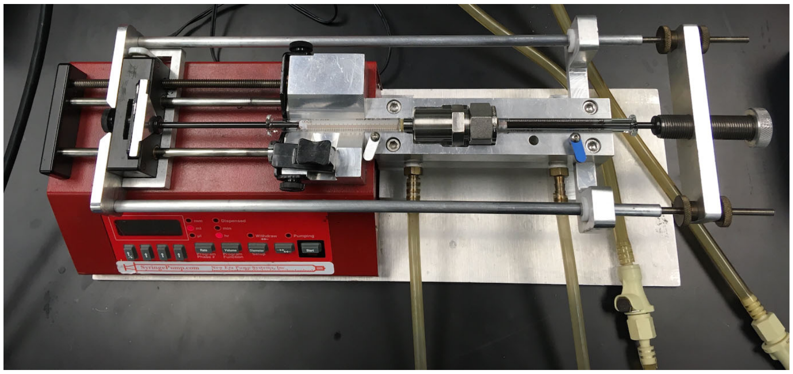

Figure S1.2 View of modified New Era Pump. The frame allows for the plungers to be pushed in either direction. The syringes are heated by a circulating bath connected to the modified heating block.

S1.2 Heating Block and Automatic Extrusion

Because this hollow metal cylinder was longer than the original cylinder from Avanti, the heating block was machined to fit this new size. The heating block was also machined to include outlets that allowed us to connect a temperature controlled Anova Scientific water bath. The tubing included quick-connects for portability as well as the ability to connect multiple extruders in series. In addition, the extrusion was automated by the use of a programmable syringe pump (New Era Pump Systems, Inc.). The programing allowed us to vary the speed and the number of passes. The automatic extruder allows for a consistent applied constant speed of extrusion that reduces leakage and hassle, and which may result in a reduced vesicle size polydispersity

S2 Contrast Matching Procedure and Scattering Lengths Table.

In order to study the phase behavior of the membrane, we had to render mixed vesicles invisible by making the mean SLD of the membrane essentially equal to the SLD of the solvent solution. Since D_2O and H_2O have very different SLDs, they can be combined to produce a solvent SLD that matches that of the vesicles, which are composed of mixture of deuterated DPPC and hydrogenated DLPC. At the contrast match point, the SLD of the vesicle matches that of the solvent, and the scattering will be that of the background. The vesicles were extruded in D_2O and H_2O at the same concentration and mixed to make four contrasts: 100% D_2O, 100% H_2O, 78.7% D_2O, 21.4% D_2O. The scattering curves obtained are shown in Figure S2.1A. Scattering was measured at 40°C where the lipid mixture is above the miscibility transition. A plot of $\sqrt{\bar{I} - \bar{I}_{bgd}}$ vs. D_2O v/v % is shown in Figure S2.1B, where $\bar{I}$ is the average of the first 2 intensity points, while $\bar{I}_{bgd}$ is the average of the last 20 points for each series sample. The error bars are smaller than the symbols used, A linear fit shows the D_2O percent where $\sqrt{\bar{I} - \bar{I}_{bgd}}$ becomes zero, corresponding to the CM point. The contrast-match percent of D_2O for our 1:1 dDPPC:DLPC mixture was found to be 55.1 ± 0.3 v/v% D_2O.

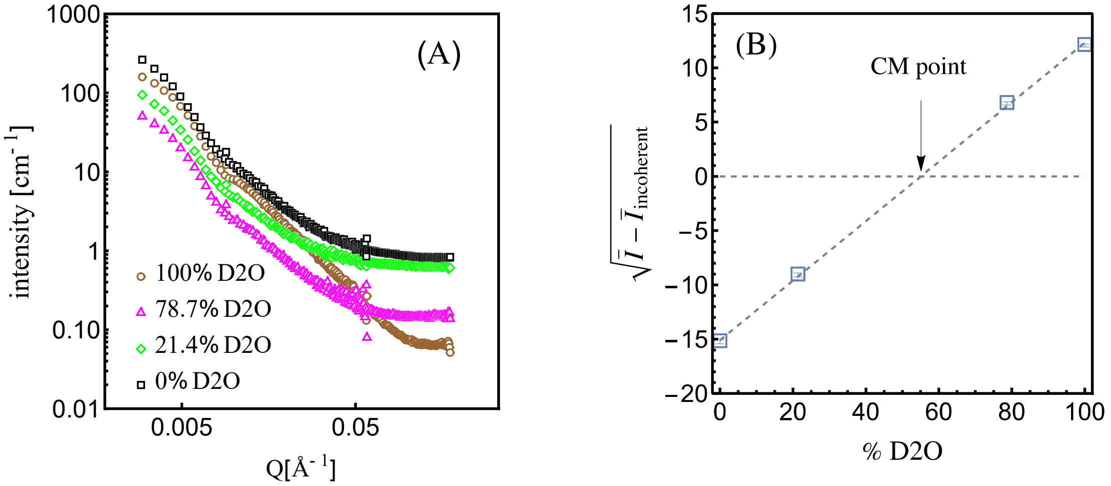

Figure S2.1 (A) I(Q) vs. Q curves for 100nm dDPPC:DLPC 1:1 vesicles in solvents with specified ratios of D_2O and H_2O at 40°C. (B) Right: A background-subtracted mean intensity vs. D_2O v/v% at four contrasts (open squares). At zero intensity, the contrast match point for dDPPC/DLPC = 1/1 was determined to be 55.1 ± 0.3 v/v% D_2O.

molecule	chemical formula	scattering length (*b*) [fm]
heavy water	D_2O	19.145
water	H_2O	−1.675
headgroup	$H_{18}C_{10}NO_8P$	60.123
tails: dDPPC	$D_{62}C_{30}$	613.135
tails: DLPC	$H_{46}C_{22}$	−25.67

Table S2.1 Scattering lengths used in the present work and determined from known nuclear scattering lengths[1].

S3 Calorimetry

Sample preparation is detailed in the Methods section. SetSoft 2000 provided by SETARAM was used to take and export DSC data. The system was equilibrated (the heatflux baseline was reported 0.00±0.01mW) before ramping the temperature. The DSC data was collected at a rate of 0.2°C/min. The raw ascii data ($mW\cdot K^{-1}$) was exported and processed in Mathematica. Heat capacities ($Jg^{-1}K^{-1}$) for the cooling of MLVs are shown in Figures 2A and 2B of the main text. In Figures S3.1A and S3.1B we show the heat capacities for MLVs during heating and 50nm in diameter SUVs during cooling, respectively.

Table S3.1 lists the corresponding onset and completion temperatures obtained from the intersection of the tangent on the heat capacity curves corresponding to the inflection point at either the onset or completion of the transition and the baseline (see Figures S3.1C and S3.1F for an example). ΔH, the latent heat ($J\cdot g^{-1}$) is given by:

$$\Delta H = \int_{T_{onset-\delta}}^{T_{completion+\delta}} c_p(T)\, dT \tag{S3.1}$$

where δ is a variable but small parameter (~ one degree) used to ensure that the whole transition is taken into account. The ΔH value for dDPPC in Table S3.1 is consistent with previously reported values for DPPC[2] showing that the enthalpy is not affected by deuteration. The ΔH value for DLPC has some variability in the literature [2-4] because of it unusual behavior[5, 6], ie, it does not have a single peak describing its transition (Figures S3.1C and S3.1F). The value we found for ΔH for the complete transition of DLPC from the fluid to the gel phase – reported in Table S3.1 – is consistent with the recent measurements by Zaytseva et al.[4]

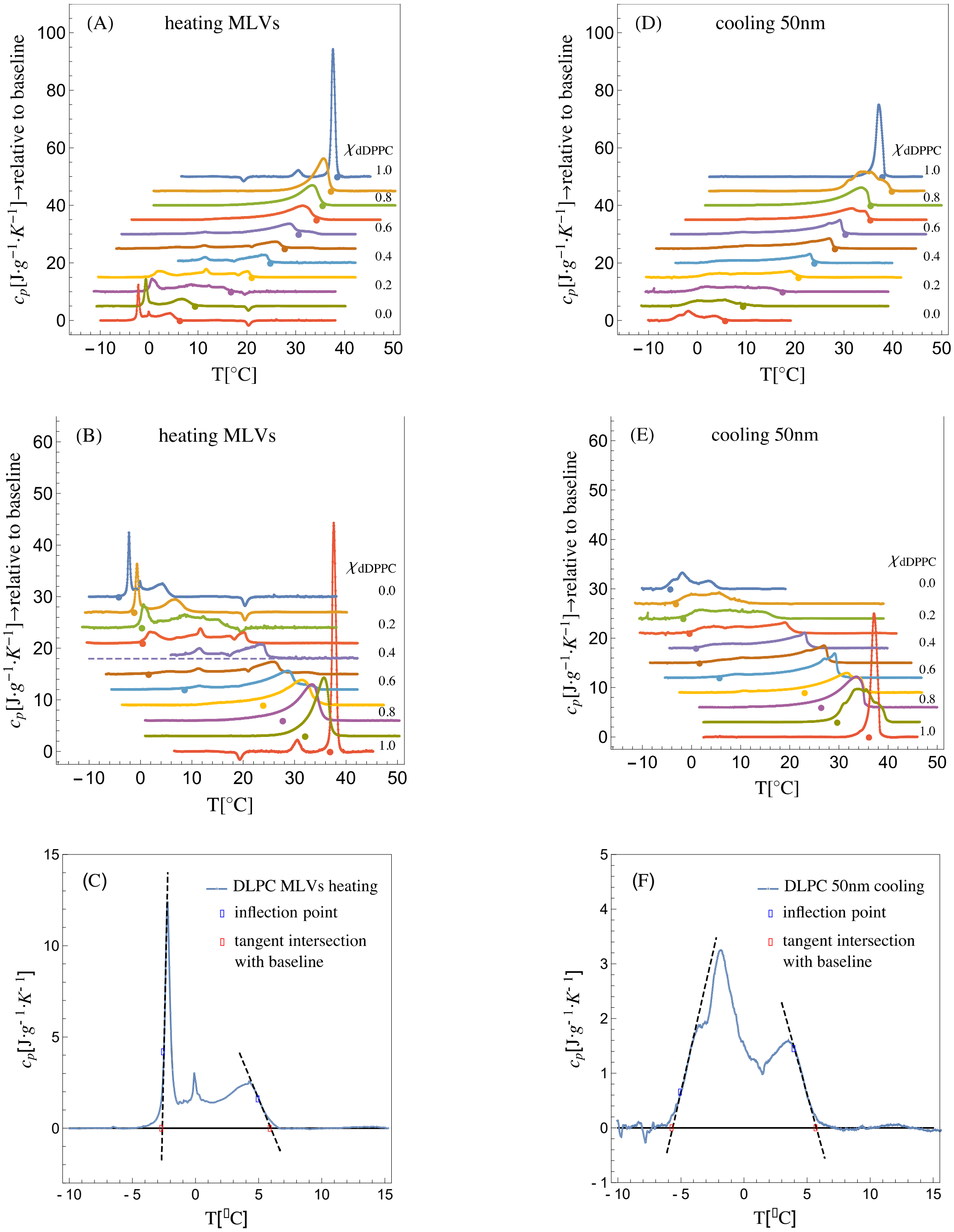

Figure S3.1 (A)-(C) Heating capacity traces for MLV for mixtures of dDPPC:DLPC. (D)-(F) Cooling heat capacity traces for 50nm SUVs for mixtures of dDPPC:DLPC. Large dots in (A) and (D) mark the onset temperature, tracing the liquidus boundary, while the large dots in (B) and in (E) mark the completion temperature, tracing the solidus boundary. (C) and (F) correspond to DLPC heat capacity traces showing the procedure to obtain transition onset and completion temperatures. The dashed lines are tangents on the c_p curve where the slope is undergoing an inflection (marked with blue dots), ie, where the slope will start to decrease (or increase) after it had been increasing (or decreasing). The transition's onset and completion temperatures are given by the intersection of this tangent with the baseline marked with red dots.

dDPPC:DLPC	Liposomes	ΔH[J·g^{-1}]	ΔH[kcal·mol^{-1}]	$T_{solidus}$[°C]	$T_{liquidus}$[°C]
0:100	MLVs heating	23. ± 0.7	3.4 ± 0.1	−2.7 ± 0.1	6.3 ± 0.3
	MLVs cooling	23. ± 2.	3.4 ± 0.3	−4.2 ± 0.3	5.8 ± 0.4
	50nm cooling	18. ± 1.7	2.7 ± 0.25	−5.5 ± 0.1	5.7 ± 0.2
10:90	MLVs heating	20. ± 0.7	3. ± 0.1	−1.2 ± 0.1	9.4 ± 0.1
	MLVs cooling	26. ± 0.7	3.8 ± 0.1	−2.2 ± 0.05	9.3 ± 0.05
	50nm cooling	24. ± 2.	3.5 ± 0.3	−3.2 ± 0.75	9.4 ± 0.05
20:80	MLVs heating	34. ± 0.34	5.1 ± 0.05	0.3 ± 0.05	17. ± 0.05
	MLVs cooling	30. ± 1	4.5 ± 0.15	−1.3 ± 0.05	16. ± 0.05
	50nm heating	29. ± 0.34	4.4 ± 0.05	−0.7 ± 0.1	18. ± 0.05
	50nm cooling	29. ± 5	4.3 ± 0.3	−1.7 ± 0.35	17. ± 0.05
30:70	MLVs heating	32. ± 1.3	4.8 ± 0.2	0.45 ± 0.05	21 ± 0.05
	MLVs cooling	33. ± 0.34	5. ± 0.05	−0.7 ± 0.2	20. ± 0.1
	50nm heating	29. ± 1.7	4.4 ± 0.25	−0.35 ± 0.15	21. ± 0.05
	50nm cooling	31. ± 0.34	4.6 ± 0.05	−0.4 ± 0.05	21. ± 0.05
40:60	MLVs heating				25. ± 0.05
	MLVs cooling	34. ± 0.7	5. ± 0.1	0.9 ± 0.1	24 ± 0.05
	50nm heating				25. ± 0.05
	50nm cooling	32. ± 0.7	4.7 ± 0.1	0.95 ± 0.25	24. ± 0.05
50:50	MLVs heating	28. ± 0.35	4.1 ± 0.05	1.6 ± 0.05	28. ± 0.05
	MLVs cooling	33. ± 0.35	4.9 ± 0.05	1.7 ± 0.1	27. ± 0.05
	50nm heating	35. ± 0.35	5.2 ± 0.05	2.3 ± 0.05	30. ± 0.05
	50nm cooling	42. ± 0.35	6.2 ± 0.05	1.7 ± 0.05	28. ± 0.15
60:40	MLVs heating	38. ± 0.35	5.7 ± 0.05	8.6 ± 0.1	31. ± 0.1
	MLVs cooling	39. ± 0.35	5.8 ± 0.05	8.1 ± 0.05	29. ± 0.05
	50nm heating	41. ± 2.7	6.1 ± 0.4	8.3 ± 0.6	32. ± 0.3
	50nm cooling	45. ± 0.35	6.8 ± 0.05	5.7 ± 0.7	30. ± 0.4
70:30	MLVs heating	48. ± 0.35	7.2 ± 0.05	24. ± 0.2	34. ± 0.05
	MLVs cooling	48. ± 0.7	7.1 ± 0.1	23. ± 0.15	33. ± 0.3
	50nm heating	48. ± 0.35	7.2 ± 0.05	23. ± 0.05	37. ± 0.05
	50nm cooling	47. ± 0.35	7.1 ± 0.05	23. ± 0.025	35. ± 0.1
80:20	MLVs heating	53. ± 0.35	7.9 ± 0.05	28. ± 0.05	36. ± 0.05
	MLVs cooling	53. ± 0.35	8. ± 0.05	28. ± 0.05	34. ± 0.05
	50nm heating	53. ± 0.35	8. ± 0.05	27. ± 0.1	37. ± 0.05
	50nm cooling	52. ± 0.35	7.8 ± 0.05	26. ± 0.3	35. ± 0.15
90:10	MLVs heating	55. ± 0.35	8.2 ± 0.05	32. ± 0.05	37. ± 0.05
	MLVs cooling	56. ± 0.35	8.4 ± 0.05	32. ± 0.05	36. ± 0.05
	50nm heating	57. ± 0.35	8.4 ± 0.05	33. ± 0.1	42. ± 0.05
	50nm cooling	61. ± 0.35	9.1 ± 0.05	30. ± 0.55	40. ± 0.05
100:0	MLVs heating	53. ± 0.35	7.9 ± 0.05	37. ± 0.05	39. ± 0.05
	MLVs cooling	55. ± 1.4	8.2 ± 0.2	36. ± 0.05	38. ± 0.05
	50nm heating	57. ± 0.35	8.5 ± 0.05	37. ± 0.1	39. ± 0.05
	50nm cooling	52. ± 0.35	7.8 ± 0.05	36. ± 0.05	38. ± 0.05

Table S3.1 Latent heats (J·g^{-1} and kcal·mol^{-1}) and the corresponding temperatures defining the solidus and liquidus boundaries for the transition from a single fluid phase to a fully solid phase upon the heating and cooling of MLVs and 50nm vesicles (and shown in Figure 2B of the main text). Error bars correspond to at least two calorimetry scans as well as instrument resolution.

S4. Compositions and Phase Fractions from Phase Diagram

As shown in Figure 2C of the main text, upon lowering the temperature below the upper miscibility or liquidus boundary, a lipid system consisting of a mixture of dDPPC and DLPC will phase separate into two phases: a solidus and a liquidus phase. The respective dDPPC to DLPC composition in each phase is obtained directly from the liquidus and solidus (lower miscibility) boundaries. That is, at a given temperature, the liquidus boundary provides the composition of the liquidus from the coordinates: (f_l, T) and the solidus boundary provides the composition of the solidus from the coordinates: (f_s, T). In addition, these coordinates provide the necessary information to extract the fraction of dDPPC molecules that are in each phase through the Lever Rule. If at a given temperature, T, f_l is the dDPPC composition of the liquidus phase and f_s is the dDPPC composition of solidus phase, and the system under study has an overall dDPPC composition given by χ^{*}_{dDPPC}, then, the fraction of dDPPC molecules in the liquidus, l, is given by:

$$l = (f_s - \chi^{*}_{dDPPC})/(f_s - f_l) \tag{S4.1}$$

This relation is called the lever rule. Here:

$$\chi^{*}_{dDPPC} = N_{dDPPC}/(N_{dDPPC} + N_{DLPC}) \tag{S4.2}$$

Correspondingly, the fraction of all dDPPC belonging to the solidus phase is simply $(1 - l)$. Here N_{dDPPC} and N_{dDPPC} are, respectively, the total number of dDPPC and DLPC molecules in the system.

Since our goal is to analyze SANS data, we need to translate these molecular fractions into volume fractions. In order to translate l into a volume fraction we will define N_l as the total number of molecules in the liquidus phase and hence:

$$l = N_l/(N_{dDPPC} + N_{DLPC}) \tag{S4.3}$$

If N_{dDPPC_l} and N_{DLPC_l} correspond to the number of dDPPC and DLPC molecules in the liquidus phase, then:

$$N_l = N_{dDPPC_l} + N_{DLPC_l} \tag{S4.4}$$

hence, the molecular fraction of dDPPC in the liquidus phase is:

$$f_l = N_{dDPPC_l}/N_l \tag{S4.5}$$

and correspondingly, the molecular fraction of DLPC in the liquidus phase is:

$$1 - f_l = N_{DLPC_l}/N_l \tag{S4.6}$$

From Eqs. S4.2 - S4.5 we can solve for N_{dDPPC_l} and N_{DLPC_l}:

$$N_{dDPPC_l} = f_l N_l = f_l l(N_{dDPPC} + N_{DLPC}) = f_l l N_{dDPPC}/\chi^{*}_{dDPPC} \tag{S4.7}$$

and

$$N_{DLPC_l} = (1 - f_l)N_l = (1 - f_l)l(N_{dDPPC} + N_{DLPC}) = (1 - f_l)lN_{dDPPC}/\chi^*_{dDPPC} \quad \text{(S4.8)}$$

Similarly, if N_{dDPPC_s} and N_{DLPC_s} correspond to the number of dDPPC and DLPC molecules in the solidus phase, then:

$$N_{dDPPC_s} = f_s(1 - l)N_{dDPPC}/\chi^*_{dDPPC} \quad \text{(S4.9)}$$

and

$$N_{DLPC_s} = (1 - f_s)(1 - l)N_{dDPPC}/\chi^*_{dDPPC} \quad \text{(S4.10)}$$

With this information (Eqs. S4.7-S4.10) we can now obtain the volume fraction of the liquidus phase, ν_l:

$$\nu_l = \frac{V_l}{V_T} = \frac{V_{dDPPC_l}N_{dDPPC_l} + V_{DLPC_l}N_{DLPC_l}}{V_{dDPPC_l}N_{dDPPC_l} + V_{DLPC_l}N_{DLPC_l} + V_{dDPPC_s}N_{dDPPC_s} + V_{DLPC_s}N_{DLPC_s}}$$

$$= \frac{V_{dDPPC_l}f_l l + V_{DLPC_l}(1-f_l)l}{V_{dDPPC_l}f_l l + V_{dDPPC_s}f_s(1-l) + V_{DLPC_l}(1-f_l)l + V_{DLPC_s}(1-f_s)(1-l)} \quad \text{(S4.11)}$$

and the solidus phase, ν_s:

$$\nu_s = 1 - \nu_l \quad \text{(S4.12)}$$

where V_{dDPPC_l}, V_{dDPPC_s}, V_{DLPC_l} and V_{DLPC_s} correspond to the molecular volumes of dDPPC and DLPC in the liquidus and solidus phases respectively.

Similarly, the dDPPC composition of the liquidus and solidus phases (f_l and f_s respectively) can also be translated into respective volume fractions of dDPPC in each phase:

$$v_{dDPPC_l} = \frac{V_{dDPPC_l}N_{dDPPC_l}}{V_{dDPPC_l}N_{dDPPC_l} + V_{DLPC_l}N_{DLPC_l}} = \frac{V_{dDPPC_l}f_l}{V_{dDPPC_l}f_l + V_{DLPC_l}(1-f_l)} \quad \text{(S4.13)}$$

$$v_{dDPPC_s} = \frac{V_{dDPPC_s}f_s}{V_{dDPPC_s}f_s + V_{DLPC_s}(1-f_s)} \quad \text{(S4.14)}$$

Finally, the molecular volume of a mixture is therefore:

$$V_T = V_L = \frac{V_{dDPPC_l}N_{dDPPC_l} + V_{DLPC_l}N_{DLPC_l} + V_{dDPPC_s}N_{dDPPC_s} + V_{DLPC_s}N_{DLPC_s}}{N_{dDPPC} + N_{DLPC}}$$

$$= V_{dDPPC_l}f_l l + V_{DLPC_l}(1 - f_l)l + V_{dDPPC_s}f_s(1 - l) + V_{DLPC_s}(1 - f_s)(1 - l) \quad \text{(S4.15)}$$

For fits of data in the two phase coexistence region at low temperature (T ≤ 18°C), the molecular volumes of DLPC and dDPPC in the solidus phase were taken to be their gel phase volumes. Hence,

$$\nu_l = \frac{V_l}{V_T} = \frac{V_{dDPPC_f}f_l l + V_{DLPC_g}(1-f_l)l}{V_{dDPPC_f}f_l l + V_{dDPPC_g}f_s(1-l) + V_{DLPC_f}(1-f_l)l + V_{DLPC_g}(1-f_s)(1-l)} \quad \text{(S4.16)}$$

$$v_{dDPPC_l} = \frac{V_{dDPPC_f}f_l}{V_{dDPPC_f}f_l + V_{DLPC_f}(1-f_l)} \quad \text{(S4.17)}$$

$$v_{dDPPC_s} = \frac{V_{dDPPC_g} f_s}{V_{dDPPC_g} f_s + V_{DLPC_g}(1-f_s)} \tag{S4.18}$$

$$V_L = V_{dDPPC_f} f_l l + V_{DLPC_f}(1-f_l) l + V_{dDPPC_g} f_s (1-l) + V_{DLPC_g}(1-f_s)(1-l) \tag{S4.19}$$

For fits of data still in the two phase coexistence region but closer to the transition temperature (24℃ ≥ T ≥ 18℃), the molecular volume of DLPC in the solidus phase was modeled to be in between its gel phase volume and its fluid phase volume using the sliding parameter α as shown by Equation 10. in the main text.

S5 Densitometry and Molecular Volumes

- densitometry

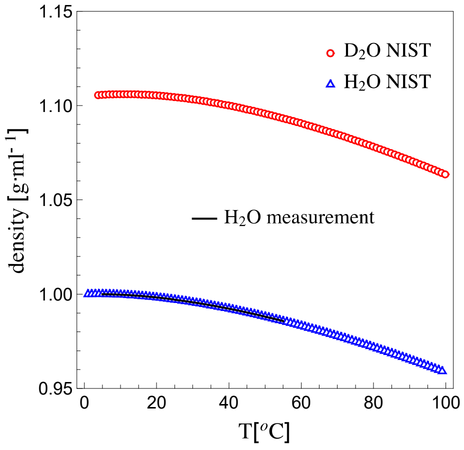

Figure S5.1 Densitometry measurements of $H_2O$and D_2O. Blue symbols for H_2O and red symbols for D_2O taken from NIST[7]. A continuous black line corresponds to our density measurement of H_2O using a density and sound velocity meter: DMA 5000 M, from Anton-Paar (Ashland, Virginia); equipment made available by the PSCM, Grenoble France.

- Molecular Volumes

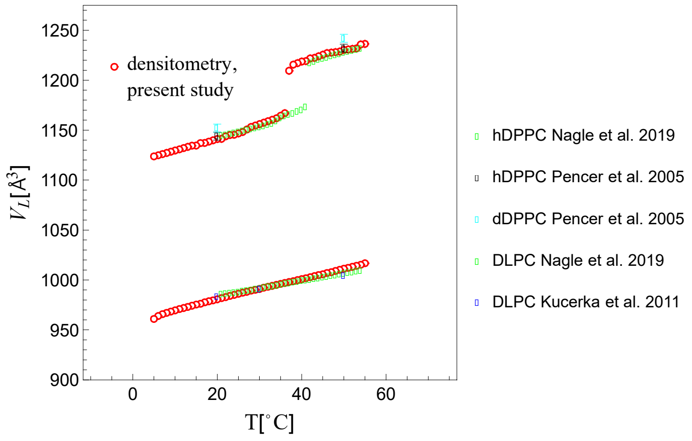

Figure S5.2 Comparison between the molecular volumes obtained in this study (red symbols) and those from Pencer et al.[8]: cyan symbol for dDPPC and black symbol for hDPPC, from Kucerka et al.[9]: blue symbols for DLPC and for Nagle et al.[10]: green symbols for both dDPPC and DLPC.

- Error in Molecular Volumes

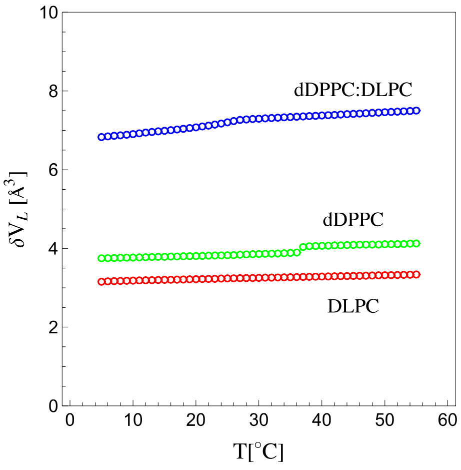

Figure S5.3 Errors for V_L , δV_L were calculated using the error in weighing (±0.0001g) and the error in the density (±0.0001g/mL).

- inverted calorimetry parameters:

inversion parameters	δ [$\times10^{-4}$cm^3·J^{-1}]	α [$\times10^{-4}$Å^3·mol·g/ °C]
dDPPC	8.5 ± 0.4	8.7 ± 0.3
1:1 dDPPC:DLPC	13.7 ± 0.6	9 ± 0.3
DLPC	15.2 ± 0.8	10.5 ± 0.3

Table S5.1 Parameters used to produce molecular volumes obtained from inverted calorimetry traces for MLVs that overlapped to those obtained from densitometry. An additional criterium was applied for DLPC to obtain α and δ: at -5°C, the molar mean of the gel volumes for dDPPC and DLPC had to coincide with the molecular volume of the 1:1 mixture.

Table S5.2 Molecular Volume equations: linear fits for dDPPC and DLPC below and above T_m, from densitometry and calorimetry; subscripts g and f refer to gel and fluid states.

$$\mathrm{dDPPC_{above\ Tm}} = \mathrm{dDPPC_f} = 1174 + 1.13\mathrm{T}\ \ \text{Å}^3$$

$$\mathrm{dDPPC_{below\ Tm}} = \mathrm{dDPPC_g} = 1118\ +\ 1.16\mathrm{T}\ \ \text{Å}^3$$

$$\mathrm{DLPC_{above\ Tm}} = \mathrm{DLPC_f} = 962\ +\ 0.98\mathrm{T}\ \ \text{Å}^3$$

$$\mathrm{DLPC_{below\ Tm}} = \mathrm{DLPC_g} = 925.2\ +\ 0.98\mathrm{T}\ \ \text{Å}^3$$

S6 SANS from hydrogenated DPPC:DLPC 50nm vesicles in D_2O

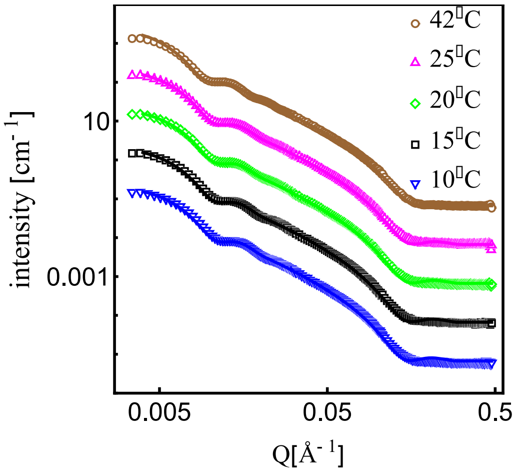

Figure S6.1 Temperature-dependent SANS measurements and corresponding fits (black lines) using a vesicle form factor for 50nm vesicles of a 1:1 ratio of hydrogenated DPPC and hydrogenated DLPC in 100% D_2O. Curves have been offset in intensity to facilitate viewing. Table S6.1 provides the fit parameters. Sample preparation protocol is detailed in the Methods section.

Table S6.1 Fit parameters from SAS view.

	42°C	25°C	20°C	15°C	10°C
bilayer thickness (Å)	36.1 ± 0.3	39.1 ± 0.3	39.7 ± 0.3	40.4 ± 0.3	40.9 ± 0.3
vesicle radius(Å)	287 ± 2	251 ± 2	249 ± 2	251 ± 2	246 ± 2
radius polydispersity	0.25	0.25	0.25	0.25	0.25

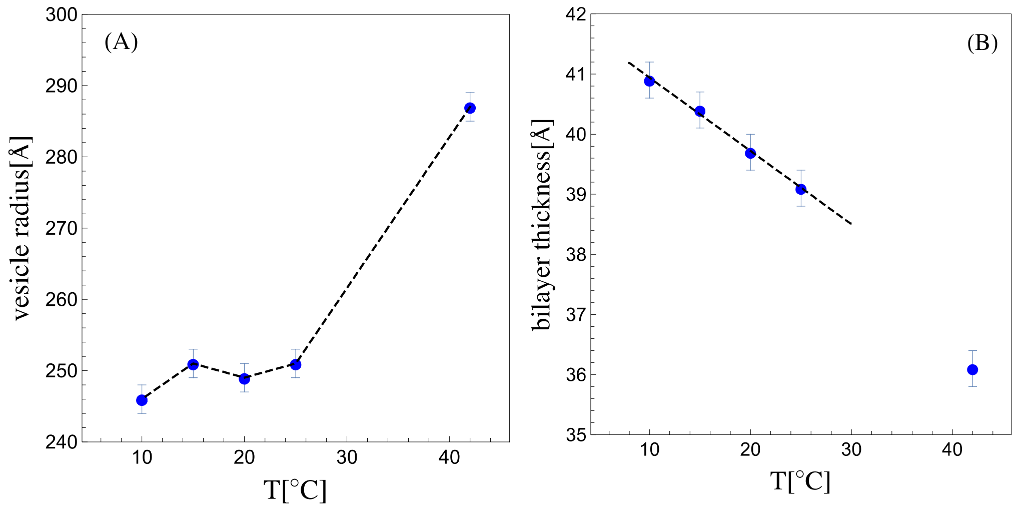

Figure S6.2 Fit parameters from SASview as a function of temperature. (A) The radius of the vesicles is seen to drop when changing the temperature from above the transition temperature to below it; at low temperatures the radius remained fairly constant. Dashed lines highlight the trends. (B) The thickness of the bilayer at 42°C is consistent with a 1:1 DPPC:DLPC membrane [9]. As with the radius, the thickness of the bilayer expands when changing the temperature from above the transition temperature to below it. In the two-phase coexistence region, there is an increase in bilayer thickness with decreasing temperature, a consistent trend with the general behavior of lipid bilayers [9] as well as binary mixtures with similar properties[11].

S7 MONSA and bead-vesicle scattering

MONSA is a powerful scattering fitting package that allows for the construction of complex/free-form structures[12]. The scattering, expressed by Eq. 1 in the main text, is replaced by a collection (sum) of many small scattering volumes (dummy atoms/beads) representing our scattering object[12]. Figure S7.1 shows a construct of a vesicle with 1260 identical beads. The contrast that each bead can take is given by the user (up to four different values). Fitting proceeds by the routine varying the contrast of some or all beads and using a simulated annealing minimization protocol[13]. Because the grid representing the position of the beads is fixed, the calculated scattering corresponds to a monodisperse structure. Indeed, many biological macromolecules, while in their tertiary/quaternary state, are known to be monodisperse. Our extruded vesicles, however, are polydisperse (~0.25). Analysis of our SANS data can proceed with this approach because the excess scattering being fitted comes only from the domains (the vesicles are contrast matched), and the domains have different shapes and sizes, and these can be incorporated into the bead-vesicle which MONSA can properly process in its scattering calculation. In MONSA we can introduce instrumental smearing through a set of instrument characteristics. These characteristics are shown in Table S7.1 below and correspond to those from the D22 SANS instrument at ILL when the data was taken.

We built bead-vesicles to fit our dDPPC:DLPC data using the vesicles' parameters in section S6, at high (42ºC) and low (20ºC) temperature. With MONSA we calculated the scattering from single SLD bead-vesicles using instrument smearing and compared them to the calculated analytical vesicle form factor scattering using SASview (also only using instrument smearing and no polydispersity), as shown in figure S7.1. The grid for the bead-vesicle had dimensions consistent with the analytical vesicle form factor in SASview: for example, the high temperature bead vesicles had a $R_{min} = 334Å$ and $R_{max} = 370Å$, which gave a bilayer thickness of 36 Å. The bead radius, r_{bead}, expected to satisfy:

$$N_{bead} \frac{4\pi}{3} r_{bead}^3 = \frac{4\pi}{3}\left(R_{max}^3 - R_{min}^3\right) \quad \text{(S7)}$$

was found to be r_{bead}=22 Å. Here N_{bead} are the number of beads (we used 1260) and R_{max} and R_{min} are the outer most and inner most points of the grid, which produced a scattering curve that overlapped with the SASview calculation as shown in Figure S7.1 on the left. If the curves between MONSA and SASview are slightly off, the bead radius can be varied slightly for the curves to overlap. For example, for the lower temperature bead-vesicle with $R_{min} = 287Å$ and $R_{max} = 323Å$, also producing a bilayer thickness of 36Å, the r_{bead} should be 20 Å however it was changed to r_{bead}=21 Å to produce the desired overlap between calculations using MONSA and SASview, as shown in Figure S7.1 on the right.

As seen in Figure S7.1, MONSA and SASview curves are congruent in the low Q ($Q < 0.1Å^{-1}$), but are clearly different in the high Q due to the discrete bead size. Hence, when fitting data with MONSA we used only the low Q. All the feature changes we wanted to capture in our contrast matched vesicles are found below $Q = 0.07Å^{-1}$ (see Figure 1 in the main text).

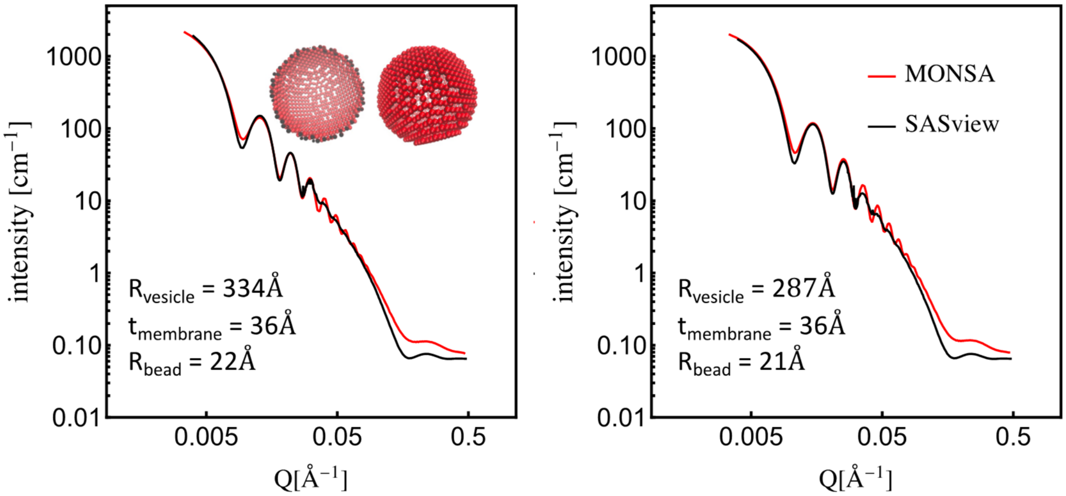

Figure S7.1 MONSA and SASview vesicle scattering calculations for a monodisperse population of vesicles as discussed in the S7 section text above. MONSA takes in bead-vesicles (top left - right). The bead-vesicle model used was built to have an approximately one-layer bead membrane as shown in the bead-vesicle cross-section (top left - left). Additional characteristics used in the calculations were a concentration at 20mg/ml, SLD contrast (hydrogenated vesicles (0.5×10^{-6}Å^{-2}) in D_2O (6.36×10^{-6}Å^{-2})), and background (0.064cm^{-1}). The instrument parameters used for MONSA is given in Table S7.1. For the SASview calculations, only the Q-dependent instrumental smearing from hydrogenated vesicle data in D_2O, and presented in S6, was used. The data used to generate the resolution function with SASview comes from 2 different set up (long collimation and long detector distance and short collimation and short detector distance to reach a large Q range) having a jump in resolution at the overlap region. This is why above 0.03 the MONSA calculation using a "high resolution" set up still displays oscillations in the form factor while analytical form factor smeared with low resolution set up in SASview does not.

Table S7.1: D22 SANS instrument at ILL

collimation characteristics	
collimation slit radius (cm)	2.57
sample diameter(cm)	1.2
collimation distance (cm)	1760.
sample−detector distance (cm)	1760.
λ (Å)	6.
$\Delta\lambda/\lambda$	0.1
pixel size (cm)	0.4
averaging error	0.

The ATSAS software suite[14], which includes MONSA, has DAMESV[15], with which one can generate bead-vesicles. The bead-vesicle is formatted as a PDB (Protein Data Bank) file required for MONSA to read it. The grid can also be built by other in-house scripts, as long as it preserves the PDB format needed to run MONSA.

S8 MONSA fitting in the two phase coexistence region

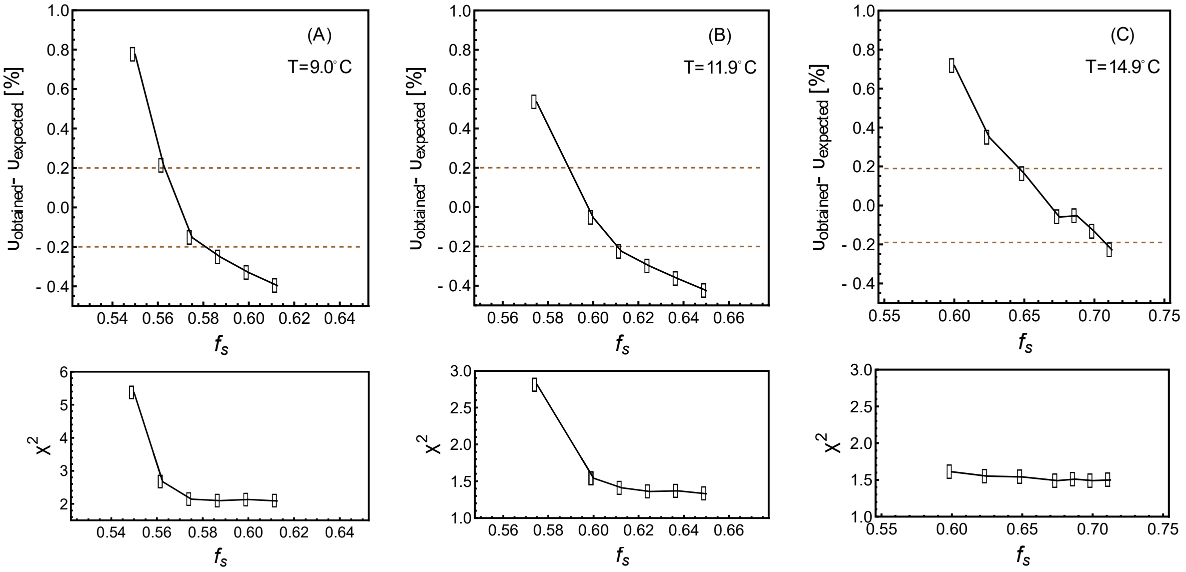

Figure S8.1. Difference in volume fraction of one phase between that expected to that obtained from the fit for three temperatures: 9℃(A), 11.9℃ (B), and 14.9℃(C). Here the liquidus is composed of only fluid phase lipids and the solidus is composed of only gel phase lipids.

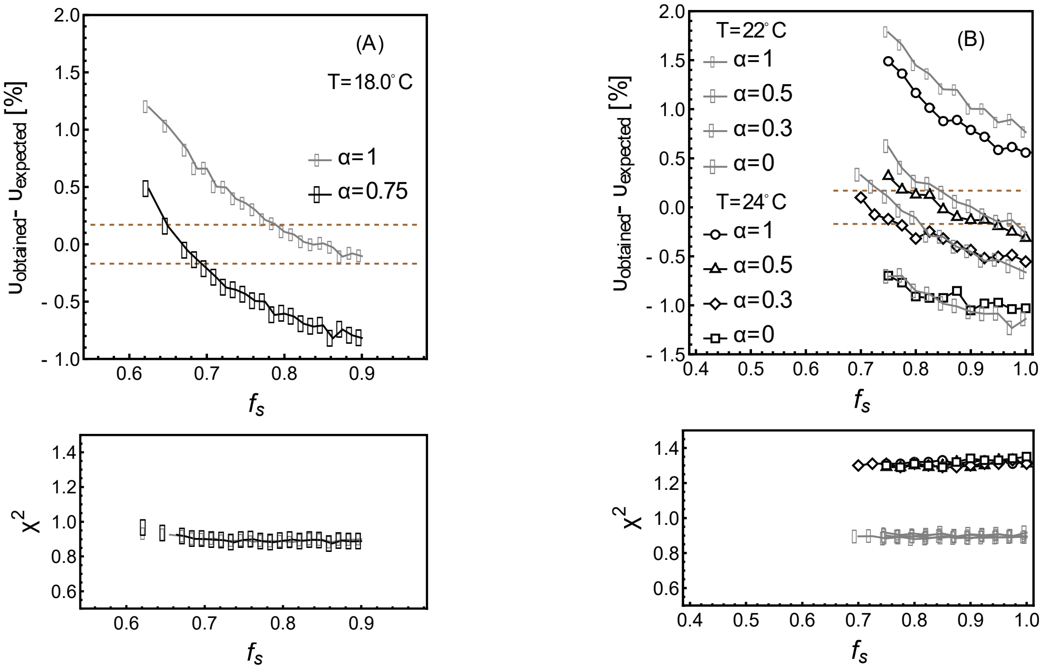

Figure S8.2. Difference in volume fraction of one phase between that expected to that obtained from the fit for three temperatures: 18℃(A), 22℃ and 24℃(B). Here the liquidus is also composed of only fluid phase lipids but the solidus is composed of dDPPC in the gel phase while DLPC attains an intermediate state between the fluid and gel state as described by the parameter α in equation 10. $\alpha = 1$ corresponds to DLPC in the gel state and $\alpha = 0$ corresponds to DLPC in the fluid state. The horizontal dashed line corresponds to the error on the volume fraction, which is ± 0.002

Table 8.1 Volume fraction of the liquidus, υ_f, as a function of temperature

T	24°C	22°C	18°C	14.9°C	11.9°C	9°C
υ_f	0.608±0.002	0.570±0.002	0.378 ± 0.002	0.320 ± 0.002	0.190 ± 0.002	0.137 ± 0.002

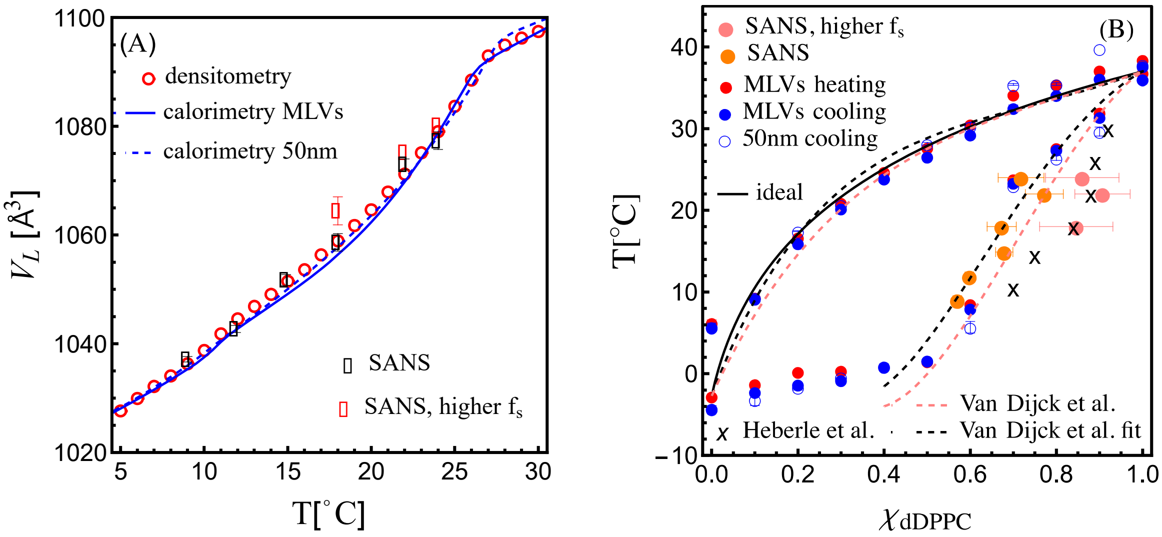

Figure S8.3 (A) Molecular volumes for the 1:1 dDPPC:DLPC mixture obtained by calorimetry (MLVs and 50nm vesicles) and densitometry (MLVs) as well as the molecular volume obtained from fits to the SANS data presented in Figure 4A of the main text (black and red symbols). The black symbols correspond to fits that reproduce the molecular volumes from densitometry and calorimetry; in these fits, the molecular volume of DLPC for T < 18°C is in the gel state but takes an intermediate volume between gel and fluid states for T ≥ 18°C . The red symbols correspond to good SANS fits as well (as shown in Figures S8.2: for α = 1 for 18°C and α = 0.5 for 22°C and 24°C) but where the larger fraction of DPPC in the solidus (i.e., a larger fs value) produce molecular volumes that do not coincide with those found by densitometry and calorimetry. (B) The corresponding phase diagram obtained by calorimetry showing the solidus boundary with points that correspond to the red and black symbols in (A). The light pink symbols correspond to the cases shown with red symbols in panel (A). The orange symbols correspond to the case shown in black symbols in panel (A). Also shown are the solidus and liquidus boundaries obtained through regular solution theory as approached by Van Dijck et al. [16]. The black dashed lines are also shown in Figure 4C and correspond to the best consistent fits to the liquidus and solidus boundaries (for the solidus boundary both the orange symbols and calorimetry points were combined) using equation 10 as described in the text. The light pink dashed lines were obtained using the values for the exponent n and the parameters $A^S$and A^L reported by Van Dijck et al. for this system.

S9 Microscopy imaging.

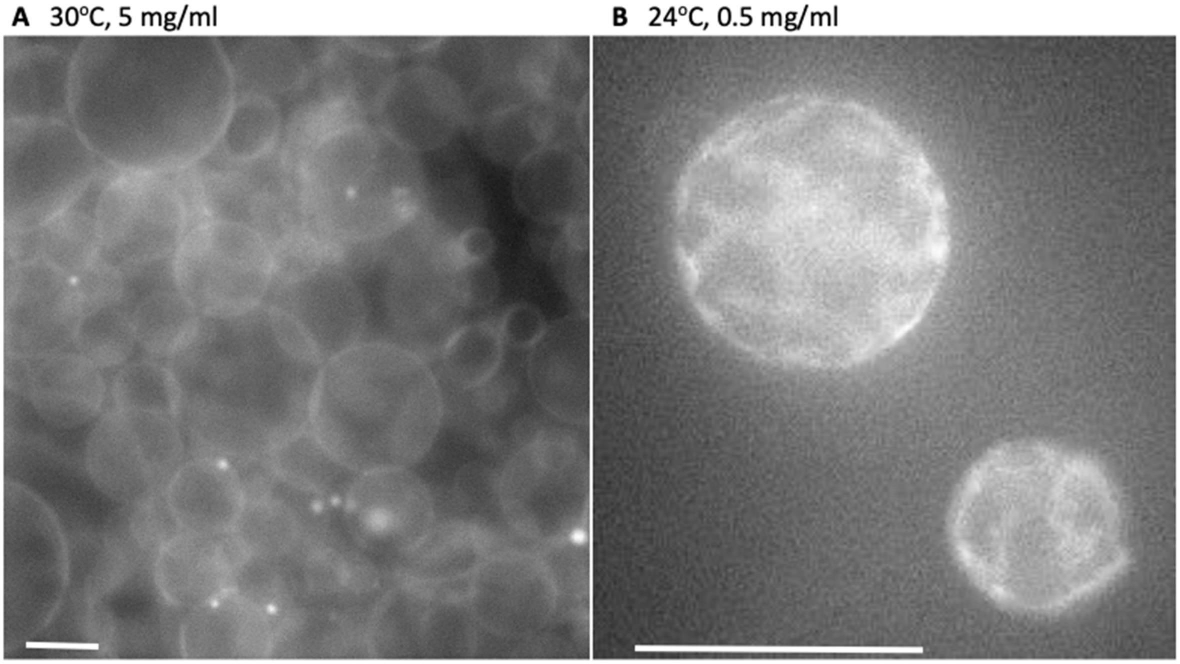

Figure S9.1 Fluorescence microscopy images of dDPPC:DLPC 1:1 GUVs. A) Above the melting temperature (T_m), the system is in the fluid phase and the dye DiI-C20:0 is uniformly distributed in the membrane. B) Below the T_m, in the two phase coexistence region, GUVs display stripes with "jagged" edges corresponding to the gel phase immersed in dark regions corresponding to the fluid phase. The scale bars correspond to 43.8 μm.

Giant unilamellar vesicles (GUVs) were prepared using the electroformation method[17]. Briefly, GUVs were prepared for a 1:1 molar ratio of DPPC:DLPC following the protocol done previously for this sample[18]; specifically, 10 mol% each of charged DLPG and DPPG (dilauroyl phosphoglycerol dipalmitoyl phosphoglycerol, respectively, purchased from Avanti Polar Lipids) was added to the binary lipid sample, which is necessary for the stability of the GUVs during electroformation. Adding charged lipids has been shown to not affect phase boundaries significantly as these are mainly driven by tail properties[19]. Fluorescent labeled amphiphilic molecules that partition into membranes, Bodipy- PC ($C_{46}H_{61}B_2F_4N_6O_8PS$, ThermoFisher Scientific) and DiI-C20:0 ($C_{60}H_{105}ClN_2O_4$, Molecular Targeting Technologies, Inc), were added at 0.1 mol% each to molar amounts of the DPPC:DLPC sample. DiI-C20:0 partitions into the solidus, DPPC-rich phase, and Bodipy-PC partitions into the liquidus, DLPC-rich phase below Tm[18]. The phases were separately illuminated, although images presented in Figure S9.1A used one illumination corresponding to the solidus phase.

GUV preparation by electroformation. The mixture of lipids and fluorophores at 4-5 mg/ml concentration in chloroform plus 5% (by volume) liquid acetonitrile was spun-cast (600 rpm for 4 minutes) onto ITO-coated glass to form a thin lipid film. After drying in vacuum (60ºC for 2 hours), a second ITO-coated glass (and a thin o-ring) was used to form a chamber with water (pre-heated to 50ºC). Subsequently, a 1 V, 10 Hz AC field was applied to this chamber for 1.5 hours at 50 ºC. After

electroformation, the GUV samples were maintained in the sealed chamber at 50 ºC for 30 minutes and then gently collected using a glass pipette.

For imaging, 10 µL of the GUV suspension was placed on a glass slide and observed using an epi-fluorescence microscope (Zeiss Observer, D1-AX10). The incident light was provided by an X-Cite series 120 Q bulb with a TEXAS RED and NBD filters (Ex/Em 565 nm/620 nm). The temperature was controlled with a Peltier heated stage plate and a Linkam controller (Linkam Scientific Instruments, United Kingdom).

S10 Correlation peak positions in the I vs Q data.

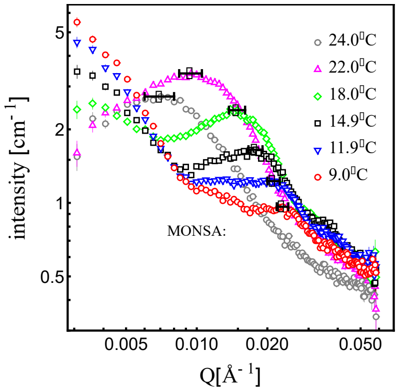

Figure S10.1 Peak positions marked with black dots. Error bars corresponds to the error in Q obtained from the instrument's configuration (wavelength spread) and measurement statistics.

S11 Round Domain Scattering

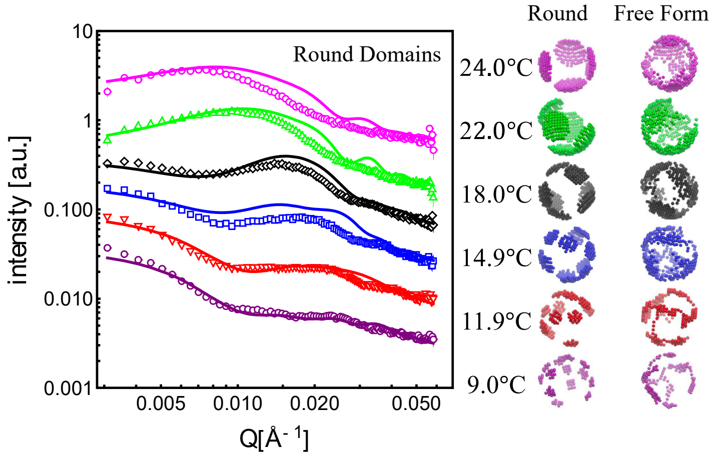

Round Domains			Free Form Domains	
$N_{domains}$	υ_f	χ^2	υ_f	χ^2
4	0.390 *solidus*	193	0.392 *solidus*	1.31
5	0.428 *solidus*	168	0.430 *solidus*	0.892
7	0.379 *liquidus*	119	0.378 *liquidus*	0.910
11	0.322 *liquidus*	295	0.320 *liquidus*	1.49
11	0.192 *liquidus*	73.9	0.190 *liquidus*	1.54
13	0.138 *liquidus*	8.12	0.137 *liquidus*	2.14

Figure S11.1 An IGOR™ script was used to generate round domains on a grid of scattering beads. Here different number of domains (each with 7 different domain arrangements) were compared to the data using MONSA to calculate the scattering curves – those with the best congruency to the data are shown on the left. Comparisons between the best round domain model configurations and the best free-form fits using bead-vesicle models are shown in the middle. The table on the right shows the number of round domains that have the best correspondence to the data, the corresponding volume fraction for the minority phase, and χ^2, a measure of the goodness between the model and the data.

[1] V.F. Sears, Neutron scattering lengths and cross sections, Neutron News 3(3) (1992) 26-37.
[2] S. Mabrey, J.M. Sturtevant, Investigation of phase transitions of lipids and lipid mixtures by sensitivity differential scanning calorimetry, Proc Natl Acad Sci U S A 73(11) (1976) 3862-6.
[3] M.R. Morrow, J.H. Davis, Calorimetric and Nuclear-Magnetic-Resonance Study of the Phase-Behavior of Dilauroylphosphatidylcholine Water, Biochim Biophys Acta 904(1) (1987) 61-70.
[4] Y.V. Zaytseva, S.V. Adichtchev, N.V. Surovtsev, Raman study of temperature-induced hydrocarbon chain disorder in saturated phosphatidylcholines, Chem Phys Lipids 230 (2020) 104926.
[5] L. Finegold, W.A. Shaw, M.A. Singer, Unusual Phase Properties of Dilauryl Phosphatidylcholine (C12pc), Chemistry and Physics of Lipids 53(2-3) (1990) 177-184.
[6] I. Hatta, S. Matuoka, M.A. Singer, L. Finegold, A new liquid crystalline phase in phosphatidylcholine bilayers as studied by X-ray diffraction, Chem Phys Lipids 69(2) (1994) 129-36.
[7] E.W.M. Lemmon, M.O.; Friend, D.G. , Thermophysical Properties of Fluid Systems, National Institute of Standards and Technology, Gaithersburg MD, 20899.
[8] J. Pencer, T. Mills, V. Anghel, S. Krueger, R.M. Epand, J. Katsaras, Detection of submicron-sized raft-like domains in membranes by small-angle neutron scattering, Eur Phys J E 18(4) (2005) 447-458.
[9] N. Kucerka, M.P. Nieh, J. Katsaras, Fluid phase lipid areas and bilayer thicknesses of commonly used phosphatidylcholines as a function of temperature, Bba-Biomembranes 1808(11) (2011) 2761-2771.
[10] J.F. Nagle, R.M. Venable, E. Maroclo-Kemmerling, S. Tristram-Nagle, P.E. Harper, R.W. Pastor, Revisiting Volumes of Lipid Components in Bilayers, J Phys Chem B 123(12) (2019) 2697-2709.
[11] R. Ashkar, M. Nagao, P.D. Butler, A.C. Woodka, M.K. Sen, T. Koga, Tuning Membrane Thickness Fluctuations in Model Lipid Bilayers, Biophysical Journal 109(1) (2015) 106-112.
[12] D.I. Svergun, K.H. Nierhaus, A map of protein-rRNA distribution in the 70 S Escherichia coli ribosome, J Biol Chem 275(19) (2000) 14432-14439.
[13] D.I. Svergun, Restoring low resolution structure of biological macromolecules from solution scattering using simulated annealing, Biophysical Journal 76(6) (1999) 2879-2886.
[14] K. Manalastas-Cantos, P.V. Konarev, N.R. Hajizadeh, A.G. Kikhney, M.V. Petoukhov, D.S. Molodenskiy, A. Panjkovich, H.D.T. Mertens, A. Gruzinov, C. Borges, C.M. Jeffries, D.I. Svergun, D. Franke, ATSAS 3.0: expanded functionality and new tools for small-angle scattering data analysis, J Appl Crystallogr 54 (2021) 343-355.
[15] D.I. Svergun, M.V. Pethoukov, MONSA manual, 2000. https://www.embl-hamburg.de/biosaxs/manuals/monsa.html#output.
[16] P.W.M. Vandijck, A.J. Kaper, H.A.J. Oonk, J. Degier, Miscibility Properties of Binary Phosphatidylcholine Mixtures - Calorimetric Study, Biochim Biophys Acta 470(1) (1977) 58-69.
[17] D.J. Estes, M. Mayer, Electroformation of giant liposomes from spin-coated films of lipids, Colloids Surf B Biointerfaces 42(2) (2005) 115-23.
[18] G.W. Feigenson, J.T. Buboltz, Ternary phase diagram of dipalmitoyl-PC/dilauroyl-PC/cholesterol: Nanoscopic domain formation driven by cholesterol, Biophysical Journal 80(6) (2001) 2775-2788.
[19] M.C. Blosser, J.B. Starr, C.W. Turtle, J. Ashcraft, S.L. Keller, Minimal effect of lipid charge on membrane miscibility phase behavior in three ternary systems, Biophys J 104(12) (2013) 2629-38.
